# Professor Willoughby D. Miller

**Published:** 1902-07

**Authors:** 


### PROFESSOR WILLOUGHBY D. MILLER.

This distinguished member of the dental profession made a hurried visit to this country during the mid-weeks of June, mainly for the purpose of receiving the degree of Doctor of Science (Sc.D.) from the University of Pennsylvania, his alma mater, at its Commencement, June 18. It is not difficult to decide in this case which has been the most honored by this, the University or Professor Miller. It is unquestionably true that an institution receives more than it gives when the world honors one of its children, and in this case the recipient of this degree has become recognized throughout the dental and medical world as one of the brilliant investigators of the nineteenth century. This degree from his own university, though much delayed, is simply a proper recognition of merit long since accepted in Germany and throughout Europe. The conferring of this degree, therefore, means more than a simple piece of parchment, for it silently emphasizes the fact that dentistry is now occupying a position that the learned institutions of the world are forced to recognize and honor. The man that learns self-respect has no difficulty in being respected, and this is true of professions. The lessons taught by Professor Miller's life are full of encouragement to the young men in dentistry, and should be carefully studied.

